# Supplementary material for: Weizmannia coagulans BC179 Alleviates Post-Alcohol Discomfort May via Taurine-Related Metabolism and Antioxidant Regulation: A Randomized, Double-Blind, Placebo-Controlled Trial
Source: Antioxidants (Basel). 2025 Aug 23;14(9):1038. doi: 10.3390/antiox14091038 (PMC12466597; doi:10.3390/antiox14091038)
Supplement: Supplementary file 1 [file antioxidants-14-01038-s001.zip › Questionnaire.pdf]

Questionnaire

The questionnaire contains 18 items, which can be used for self-assessment or as items for inquiry. Generally, the time taken to complete the questionnaire does not exceed 15 minutes. The advantage of this form is that it can test a large number of people in a relatively short time.

Investigators should note: ① Many alcohol addicts often hide their problems and are unwilling to give truthful answers; ② For those with difficulties in reading or comprehension, explanations should be given in plain language; ③ When using the questionnaire, the subjects should be in a sober state; if they are intoxicated or in a withdrawal reaction state, their answers may be invalid. For specific instructions, please refer to the questionnaire. Subjects are required to carefully consider each question, circle the corresponding item, with only one answer per question, and every question must be answered.

Scoring Method

The scoring for the 18 items is as follows. For example, Item 1: "How much did you drink the last time you drank?"

a. Not excessive or just enough: 0 points
b. Got drunk: 1 point
c. Got extremely drunk: 2 points

And so on. The total hangover symptom score is the sum of all item scores (0-36 points).

Explanation:

The sum of the scores of all items in the Hangover Questionnaire is called the total score. The minimum is 0 points and the maximum is 36 points, which can be divided into five levels. The clinical significance of each level is as follows:

0 points: No hangover symptoms.
1-8 points: Low hangover level, mild symptoms.
9-15 points: Moderate hangover.
16-23 points: High hangover level, mild symptoms.
24-36 points: Severe hangover.

Advantages of the Hangover Questionnaire:

This scale has a theoretical basis, formulated based on the concept of alcohol hangover, and is associated with the multi-factor theory of drinking and alcohol abuse.
It can provide quantitative indicators of the severity of alcohol hangovers.
It is relatively concise, convenient, and low-cost.
It has good consistency and validity.
It can be used for research and clinical diagnosis.

Disadvantages of the Hangover Questionnaire:

Due to the obvious content of the items, subjects may intentionally cover up their problems.
Due to large individual differences, scores may be misinterpreted, so the severity of a hangover cannot be diagnosed solely based on the score.
Further research is needed to provide treatment strategies for clinicians based on scores.

Instructions

Please read each question and the corresponding answers carefully, select the answer that best matches your actual situation, and circle the corresponding answer.
Please think carefully and answer all questions as soon as possible.
If you have any questions you don't understand, please ask the investigator.

Name: Age: Gender:

Survey Date: Year Month Day

The following questions refer to your actual situation since the last time you drank.

How much did you drink the last time you drank?

A. Not excessive or just right

B. Got drunk

C. Got extremely drunk

Did you experience a headache?

A. No

B. Sometimes

C. Often

Did you experience nausea?

A. No

B. Sometimes

C. Often

Did you experience dizziness?

A. No

B. Sometimes

C. Often

Did you experience emotional excitement?

A. No

B. Sometimes

C. Often

Did you experience thirst?

A. No

B. Sometimes

C. Often

Did you experience fatigue?

A. No

B. Sometimes

C. Often

Did you experience nervousness?

A. No

B. Sometimes

C. Often

Did you experience paleness?

A. No

B. Sometimes

C. Often

Did you experience tremors?

A. No

B. Sometimes

C. Often

Did you experience vomiting?

A. No

B. Sometimes

C. Often

Did you experience heartburn?

A. No

B. Sometimes

C. Often

Did you experience unsteady gait?

A. No

B. Sometimes

C. Often

Did you experience decreased appetite?

A. No

B. Sometimes

C. Often

Did you experience insomnia?

A. No

B. Sometimes

C. Often

Did you experience hand tremors?

A. No

B. Sometimes

C. Often

Did your blood pressure increase or decrease?

A. No

B. Sometimes

C. Often

Did you experience symptoms of acute anxiety, irritability, hypersensitivity, depression, or guilt?

A. No

B. Sometimes

C. Often
