# Supplementary material for: Weizmannia coagulans BC179 Alleviates Post-Alcohol Discomfort May via Taurine-Related Metabolism and Antioxidant Regulation: A Randomized, Double-Blind, Placebo-Controlled Trial
Source: Antioxidants (Basel). 2025 Aug 23;14(9):1038. doi: 10.3390/antiox14091038 (PMC12466597; doi:10.3390/antiox14091038)
Supplement: Supplementary file 1 [file antioxidants-14-01038-s001.zip › Supplementary Table S1.pdf]

Table S1.

Projects	Placebo group (n=30)		BC99 group (n=30)		
	Mean	SD	Mean	SD	*p* value
Age	43.60	11.31	42.87	11.15	0.801
Sex(M/F)	30/0		29/1		0.313
**Metabolic characteristics**					
Height (cm)	173.4	5.02	171.03	5.38	0.084
Weight (Kg)	72.85	9.15	72.63	7.09	0.919
BMI (Kg/m^2)	24.24	2.89	24.87	2.68	0.382
**Liver function indicators**					
ALT (U/L)	40.60	22.81	40.50	23.73	0.990
AST (U/L)	35.82	11.13	40.17	21.38	0.392
γ-GT (U/L)	60.79	22.49	61.08	51.06	0.979
TBil (μmol/L)	12.42	3.48	14.03	3.11	0.073
**Renal function indicators**					
BUN (mmol/L)	5.92	0.88	6.16	0.78	0.393
Cr (μmol/L)	73.67	10.20	70.41	10.20	0.325
UA (mmol/L)	393.67	75.41	406.74	71.13	0.500
